# Commentary: Incubation and Intuition in Creative Problem Solving

**DOI:** 10.3389/fpsyg.2016.01807

**Published:** 2016-11-16

**Authors:** Yuan Yuan, Wangbing Shen

**Affiliations:** ^1^School of Psychology and Lab of Cognitive Neuroscience, Nanjing Normal UniversityNanjing, China; ^2^School of Rehabilitation Science, Nanjing Normal University of Special EducationNanjing, China; ^3^Institute of Applied Psychology and School of Public Administration, Hohai UniversityNanjing, China

**Keywords:** incubation, creative insight, dual process theories, mental impasse, intuition

There is a peculiar psychological phenomenon referred to as *incubation*; it is regarded as one of Wallas 's (1926) four stages of creativity and is defined as a departure from an ongoing unsolved problem that can lead to, or make more likely, the sudden or insightful solution of a difficult problem. Although the existence of incubation effects has long been recognized, the mechanism underlying incubation remains unclear.

Gilhooly (2016) concluded that the Unconscious Work hypothesis (UWH) provided a better explanation of incubation than the Intermittent Conscious Work (ICW) and Beneficial Forgetting (BF) hypotheses, and noted that incubation was an interesting finding that warranted further investigation. We agree with Gilhooly (2016) that the UWH provides an important account of incubation effect in divergent thinking, but here we consider a complementary perspective that deals primarily with the dual-process theory (DPT) of incubation during creative thinking, particularly convergent thinking or insight problem solving (insightful incubation). DPT has been applied to a range of cognitive processes such as judgment/decision-making (Greene, 2007), reasoning (Evans, 2008), and creativity (Lin and Lien, 2013; Weisberg, 2015).

As Gilhooly (2016) noted, the kind of convergent thinking required to solve insight tasks is just as important to creative thinking as divergent thinking. Divergent thinking processes are ill-defined, relatively unconstrained, and generate multiple solutions whereas convergent thinking relies on speed, accuracy, logic, and the capacity to recognize the familiar quickly in order to identify the single solution to a well-defined problem (Cropley, 2006; Chermahini and Hommel, 2012). To solve divergent thinking tasks such as the alternative uses task the solver only needs to switch freely between perspectives and select one subdominant characteristic of the common object (Chrysikou and Thompson-Schill, 2011; Gilhooly, 2016); he/she can easily generate infrequent uses of this object. In contrast, solving an insight problem with a defined solution requires one to understand the logic underlying the solution, to restructure one's initial representation of the problem, relax certain constraints, and overcome a set-related impasse through failure-driven learning.

There is mounting evidence that solving most convergent thinking tasks, in particular, insight problems, depends on conscious cognitive control, including working memory, inhibition, updating, and switching (Koppel and Storm, 2014; Gilhooly et al., 2015; Lv, 2015). It is often assumed that the process of overcoming an impasse, or shifting cognitive set—commonly regarded as the defining feature of insight—happens during insightful incubation (Christensen and Schunn, 2005; Zhan et al., 2015). The process of set-shifting or overcoming an impasse is a controlled, internally driven shift in strategy which has been linked to activation of the medial prefrontal region (Schuck et al., 2015) and to an error-related negative potential in the same region (Bartholow et al., 2005). The controlled inhibition of irrelevant associations or inappropriate thought patterns is required to overcome an impasse or shift cognitive set during insightful incubation (Koppel and Storm, 2014; Lv, 2015). Replacing inappropriate thoughts with appropriate ideas, which is considered essential to the process of overcoming an impasse, relies on memory updating (Jones, 2003). Gilhooly (2016, p.7) stated that “unconscious work is parallel, inexact and divergent while conscious thought is serial, exact, and convergent.”

The UWH cannot accommodate findings on insightful incubation effects (Zhong et al., 2008) because it describes incubation solely in terms of unconscious processes. A DPT-based model of incubation in terms of unconscious and conscious processing does not suffer from this problem. Our DPT model of incubation accepts, like the UWH, that unconscious processes are critical to incubation, but posits that there are two systems of thinking: system 1, which is characterized by rapid, implicit, parallel and unconscious processes and system 2, which relies on slow, explicit, sequential, conscious processes (Evans, 2008). Also, the DPT believes the conscious process underlying incubation is not intermittent and always goes with unconscious processes during the whole incubation period. Moreover, the DPT of incubation extends the UWH in three critical ways.

Firstly, the DPT proposes that both conscious and the unconscious processes are critical to incubation, whereas the UWH does not assign conscious processes any role in incubation. The UWH cannot explain why filling the incubation period with a light cognitive demand task has more incubation effect than filling it with rest or a demanding task. The DPT explains this by assuming that performing an undemanding task ensures that both unconscious and conscious processes are recruited during incubation, whereas resting makes incubation an entirely conscious process and performing a demanding task makes it entirely unconscious because cognitive resources are depleted by the task (Sio and Ormerod, 2009). Importantly, the DPT can resolve the conflicts between the UWH and other incubation theories/findings like the BF theory or the explicit-implicit interaction model (Hélie and Sun, 2010). Moreover, Zhong et al. (2008) found that unconscious thought can increase solution accessibility but may reduce the actual solution quantity when compared with conscious thought; this result is inconsistent with the UWH but is accommodated by the DPT. Sio and Ormerod's (2009) meta-analysis on incubation studies concluded that both unconscious and conscious processes were involved.

Secondly, the DPT can account for both insightful incubation and certain incubation-related phenomena in divergent thinking, such as the superiority of immediate incubation. Gilhooly (2016) argued that the difference between immediate and delayed incubation is that one of the two only involves unconscious processing whereas the other also involves conscious processing (for details, see Gilhooly et al., 2012). Given that initiating a “dual-task” delayed incubation (involving the target task and interpolated task) relative to a “single-task” immediate incubation will cost more cognitive resources, the conscious and unconscious processes could both be involved in immediate incubation but only the unconscious process in delayed incubations due to an additional depletion in cognitive resources for the interpolated task during an immediate incubation.

Finally, the DPT can explain the inverted-U shaped relationship between unconscious thoughts and creative performance (Yang et al., 2012) and the timing of the unconscious thought advantage during the incubation process. Only when the duration deliberation is moderately long, neither too long nor too short, can the unconscious thought offer significant advantages over conscious thought (Yang et al., 2012); over short or long deliberation periods, the creative output of conscious thought surpasses that of unconscious thought.

## Author contributions

WS and YY discussed the study and co-wrote this comment. WS and YY revised this comment according to the reviewers' comments. All authors have read and approved the final manuscript.

### Conflict of interest statement

The authors declare that the research was conducted in the absence of any commercial or financial relationships that could be construed as a potential conflict of interest.

## References

[B1] Bartholow B. D. Pearson M. A. Dickter C. L. Sher K. J. Fabiani M. Gratton G. (2005). Strategic control and medial frontal negativity: beyond errors and response conflict. Psychophysiology 42, 33–42. 10.1111/j.1469-8986.2005.00258.x 15720579

[B2] Chermahini S. A. Hommel B. (2012). Creative mood swings: divergent and convergent thinking affect mood in opposite ways. Psychol. Res. 76, 634–640. 10.1007/s00426-011-0358-z 21695470 PMC3412079

[B3] Christensen B. T. Schunn C. D. (2005). Spontaneous access and analogical incubation effects. Creat. Res. J. 17, 207–220. 10.1080/10400419.2005.9651480

[B4] Chrysikou E. G. Thompson-Schill S. L. (2011). Dissociable brain states linked to common and creative object use. Hum. Brain Mapp. 32, 665–675. 10.1002/hbm.21056 20533561 PMC3846690

[B5] Cropley A. (2006). In praise of convergent thinking. Creat. Res. J. 18, 391–404. 10.1207/s15326934crj1803_13

[B6] Evans J. S. B. (2008). Dual-processing accounts of reasoning, judgment, and social cognition. Annu. Rev. Psychol. 59, 255–278. 10.1146/annurev.psych.59.103006.093629 18154502

[B7] Gilhooly K. J. (2016). Incubation and intuition in creative problem solving. Front. Psychol. 7:1076. 10.3389/fpsyg.2016.01076 27499745 PMC4956660

[B8] Gilhooly K. J. Ball L. J. Macchi L. (2015). Insight and creative thinking processes: routine and special. Think. Reason. 21, 1–4. 10.1080/13546783.2014.966758

[B9] Gilhooly K. J. Georgiou G. J. Garrison J. Reston J. D. Sirota M. (2012). Don't wait to incubate: immediate versus delayed incubation in divergent thinking. Mem. Cognit. 40, 966–975. 10.3758/s13421-012-0199-z 22382649

[B10] Greene J. D. (2007). Why are VMPFC patients more utilitarian? A dual-process theory of moral judgment explains. Trends Cog. Sci. 11, 322–323. 10.1016/j.tics.2007.06.004 17625951

[B11] Hélie S. Sun R. (2010). Incubation, insight, and creative problem solving: a unified theory and a connectionist model. Psychol. Rev. 117, 994–1024. 10.1037/a0019532 20658861

[B12] Jones G. (2003). Testing two cognitive theories of insight. J. Expl Psychol: Learn. Mem. Cogn. 29:1017. 10.1037/0278-7393.29.5.1017 14516232

[B13] Koppel R. H. Storm B. C. (2014). Escaping mental fixation: incubation and inhibition in creative problem solving. Memory 22, 340–348. 10.1080/09658211.2013.789914 23607286

[B14] Lin W. L. Lien Y. W. (2013). The different role of working memory in open-ended versus closed-ended creative problem solving: a dual-process theory account. Creat. Res. J. 25, 85–96. 10.1080/10400419.2013.752249

[B15] Lv K. (2015). The involvement of working memory and inhibition functions in the different phases of insight problem solving. Mem. Cognit. 43, 709–722. 10.3758/s13421-014-0498-7 25604643

[B16] Schuck N. W. Gaschler R. Wenke D. Heinzle J. Frensch P. A. Haynes J. D.. (2015). Medial prefrontal cortex predicts internally driven strategy shifts. Neuron 86, 331–340. 10.1016/j.neuron.2015.03.015 25819613 PMC4425426

[B17] Sio U. N. Ormerod T. C. (2009). Does incubation enhance problem solving? A meta-analytic review. Psychol. bull. 135, 94–120. 10.1037/a0014212 19210055

[B18] Wallas G. (1926). The Art of Thought. New York, NY: Harcourt Brace Jovanovich.

[B19] Weisberg R. W. (2015). Toward an integrated theory of insight in problem solving. Think. Reason. 21, 5–39. 10.1080/13546783.2014.886625

[B20] Yang H. Chattopadhyay A. Zhang K. Dahl D. W. (2012). Unconscious creativity: when can unconscious thought outperform conscious thought? J. Consum. Psychol. 22, 573–581. 10.1016/j.jcps.2012.04.002

[B21] Zhan H. J. Liu C. Shen W. B. (2015). Neural basis of creative thinking during four stages. Adv. Psychol. Sci. 23, 213–224. 10.3724/SP.J.1042.2015.00213

[B22] Zhong C. B. Dijksterhuis A. Galinsky A. D. (2008). The merits of unconscious thought in creativity. Psychol. Sci. 19, 912–918. 10.1111/j.1467-9280.2008.02176.x 18947357

